# Multiple invasions of an infectious retrovirus in cat genomes

**DOI:** 10.1038/srep08164

**Published:** 2015-02-02

**Authors:** Sayumi Shimode, So Nakagawa, Takayuki Miyazawa

**Affiliations:** 1 Laboratory of Signal Transduction, Department of Cell Biology, Institute for Virus Research, Kyoto University, 53 Shogoin-Kawaharacho, Sakyo-ku, Kyoto 606-8501, Japan; 2 Department of Molecular Life Science, Tokai University School of Medicine, 143 Shimokasuya, Isehara, Kanagawa 259-1193, Japan

## Abstract

Endogenous retroviruses (ERVs) are remnants of ancient retroviral infections of host germ-line cells. While most ERVs are defective, some are active and express viral proteins. The RD-114 virus is a replication-competent feline ERV, and several feline cell lines produce infectious RD-114 viral particles. All domestic cats are considered to have an ERV locus encoding a replication-competent RD-114 virus in their genomes; however, the locus has not been identified. In this study, we investigated RD-114 virus-related proviral loci in genomes of domestic cats, and found that none were capable of producing infectious viruses. We also found that all domestic cats have an RD-114 virus-related sequence on chromosome C2, termed RDRS C2a, but populations of the other RDRSs are different depending on the regions where cats live or breed. Our results indicate that RDRS C2a, the oldest RD-114-related provirus, entered the host genome before an ancestor of domestic cats started diverging and the other new RDRSs might have integrated into migrating cats in Europe. We also show that infectious RD-114 virus can be resurrected by the recombination between two non-infectious RDRSs. From these data, we conclude that cats do not harbor infectious RD-114 viral loci in their genomes and RD-114-related viruses invaded cat genomes multiple times.

Endogenous retroviruses (ERVs) are retroviruses which have been integrated into the host genome of germ-line cells, comprising approximately 10% of the host genome in mammals^1^. ERVs behave as host genes and are transmitted from parents to offspring in a Mendelian manner. Although most ERVs are inactivated through the accumulation of mutations and deletions, leading to an introduction of stop codons within coding sequences, some ERVs are active and have the potential to produce infectious viral particles.

The RD-114 virus is a replication-competent feline ERV^2^ and several feline cell lines produce infectious RD-114 viral particles^3^. The RD-114 virus was first isolated from human rhabdomyosarcoma (RD) cells during passages through fetal cat brains and was mistakenly regarded as the first human retrovirus^4^. Subsequently, it was revealed that cellular RNA from cats contain sequences homologous to RD-114 viral RNA^5^,^6^, leading to the discovery of RD-114 virus-related proviral sequences in the cat genome^7^,^8^,^9^,^10^. In addition, several groups reported that infectious RD-114 viral particles were produced, either spontaneously or after chemical induction, from cells originating from domestic cats^11^,^12^,^13^. From these data, it was concluded that the RD-114 virus was an active ERV of cats, and not an exogenous human retrovirus.

Several research groups have searched for the active RD-114 proviral loci^2^,^14^,^15^. Spodick *et al*. identified thirteen RD-114 virus-related clones, consisting of conserved *gag*-*pol* and unrelated *env* regions from six domestic cat genomes^14^. At the same time, another group identified nine endogenous RD-114 virus-related sequence clones representing at least eight distinct loci from cat DNA libraries^2^. Although the *gag*-*pol* region of the clones was conserved, the *env* region displayed considerable divergence from the replication-competent RD-114 virus clone derived from human RD cells, both in size and sequence homology, coinciding with previous results. In their followup study, feline chromosomes which contain RD-114 virus-related segments were identified with variation of distinct viral segments among individual cats^15^. These two groups identified the restriction endonuclease-digested genome fragments corresponding to fragments of the infectious RD-114 virus clone in cat genomic DNA and concluded that the active infectious RD-114 viral locus may be a single copy. The former group detected a 4.8-kb *Pst*I-digested *pol*-*env* fragment in all cats genomic DNAs examined^14^, and the latter group detected a 1.0-kb *Bam*HI-digested *gag* fragment^2^,^15^. Although the latter fragment was found to be located on chromosome B3^15^, the locus of the former fragment was not identified. Further it has not been confirmed whether both of them were present on the same single locus as the complete full-length provirus. After these studies, a new endogenous type C retrovirus named *Felis catus* endogenous retrovirus (FcEV) which consists of the RD-114 virus-related *gag*-*pol* gene and unrelated *env* gene was discovered in all domestic cats genomes examined^16^. They reported that there were fifteen to twenty FcEVs in domestic cat genomes and suggested that all or most RD-114 virus-related sequences (RDRSs) possessing unrelated *env* genes are more likely to be FcEV integrations^16^,^17^. Therefore, as mentioned above, the RD-114 proviral locus encoding an infectious RD-114 virus has not been identified.

In this study, we searched for loci encoding infectious RD-114 virus. Contrary to our expectations, we could not find any RDRSs capable of producing infectious RD-114 virus, but discovered that a RD-114 virus-related virus (RDRV) nearly identical to the RD-114 virus can be produced by recombination between two defective RD-114 virus-related proviruses. We also noticed that most RDRSs have not been fixed in domestic cat genomes, suggesting multiple invasions of cat genomes by the ancient RDRV. Our study sheds light on host-virus interactions and provides novel genetic markers of domestic cats available for tracing footsteps of cats.

## Results

### Characterization of RDRS loci in the genomes of domestic cats

**Figure 1:**
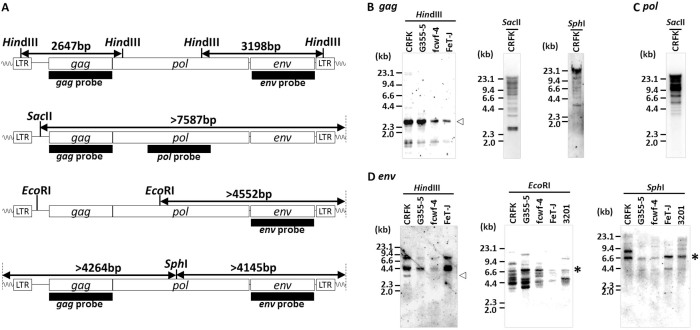
Detection of RD-114 proviruses in the genomes of feline cell lines by Southern blotting analyses. (A) Restriction endonuclease maps of an infectious molecular clone of RD-114 virus, termed pSc3c. Probes for *gag*, *pol* and *env* genes are shown as bars. (B–D) Southern blotting analyses of several feline cell lines. Genomic DNAs were digested with *Hin*dIII, *Sac*II, *Eco*RI or *Sph*I and then subjected to Southern blotting analyses using *gag* (B), *pol* (C) and *env* (D) probes. *Hin*dIII-digested fragments corresponding to pSc3c are shown by white arrowheads. Asterisks indicate RD-114 viral *env* locus detected in common in all cell lines examined.

**Figure 2:**
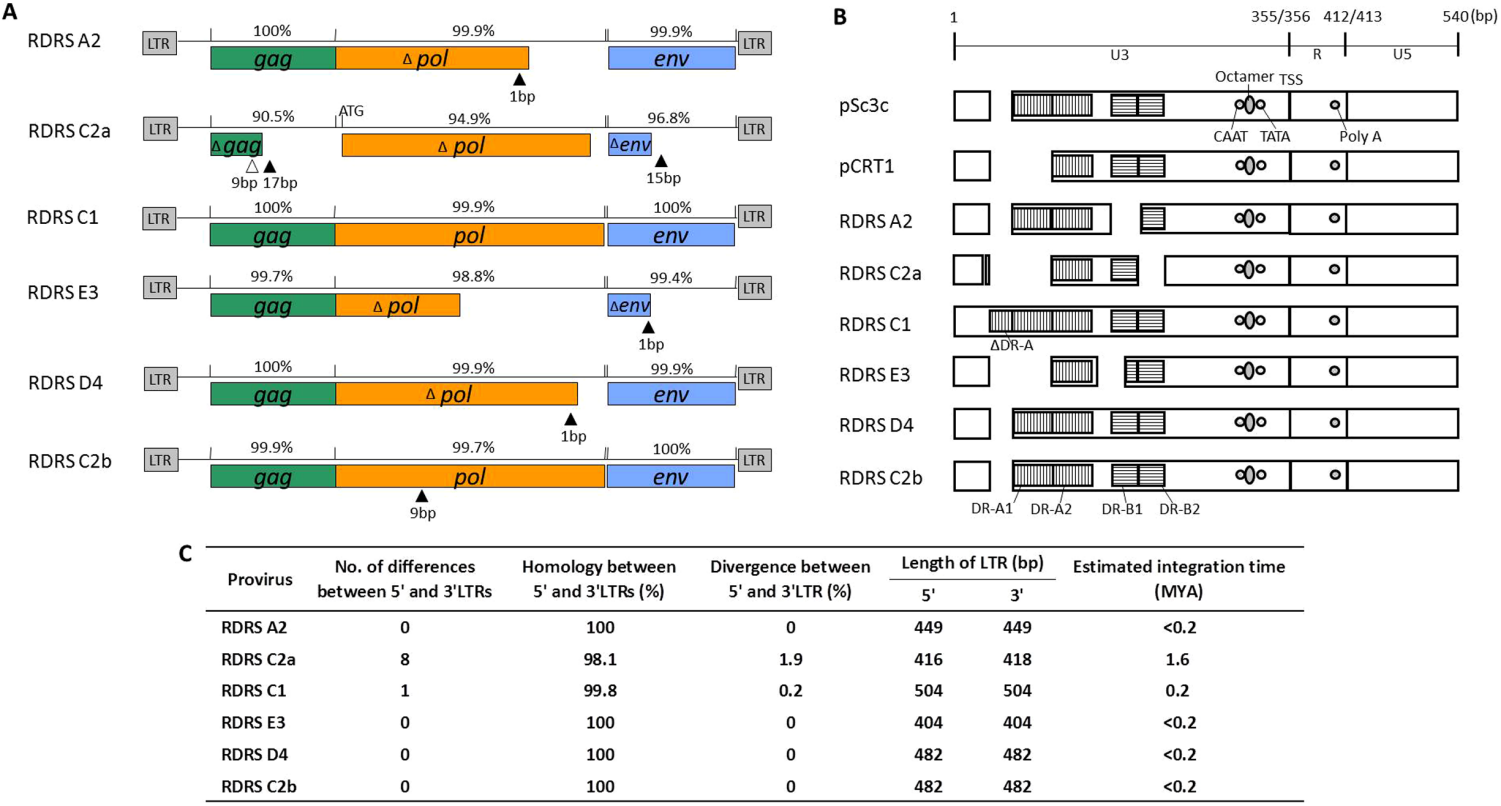
Structures of RDRSs. (A) Structures of the genomes of full length RDRSs. Open triangle indicates a deletion of nucleotides and filled triangle indicates an insertion of nucleotides. Green, orange and blue boxes indicate *gag*, *pol* and *env* ORFs, respectively. Numbers (%) above the diagrams indicate homologies with RD-114 infectious clones in each gene. (B) Schematic LTR structures of RDRSs and RD-114 virus clones, pSc3c and pCRT1. DR-A, direct repeat A; DR-B, direct repeat B; CAAT, CAAT box; TATA, TATA box; TSS, transcription start site; poly A, poly A signal. (C) Features of LTRs of RDRS proviruses and estimated integration time.

**Figure 3:**
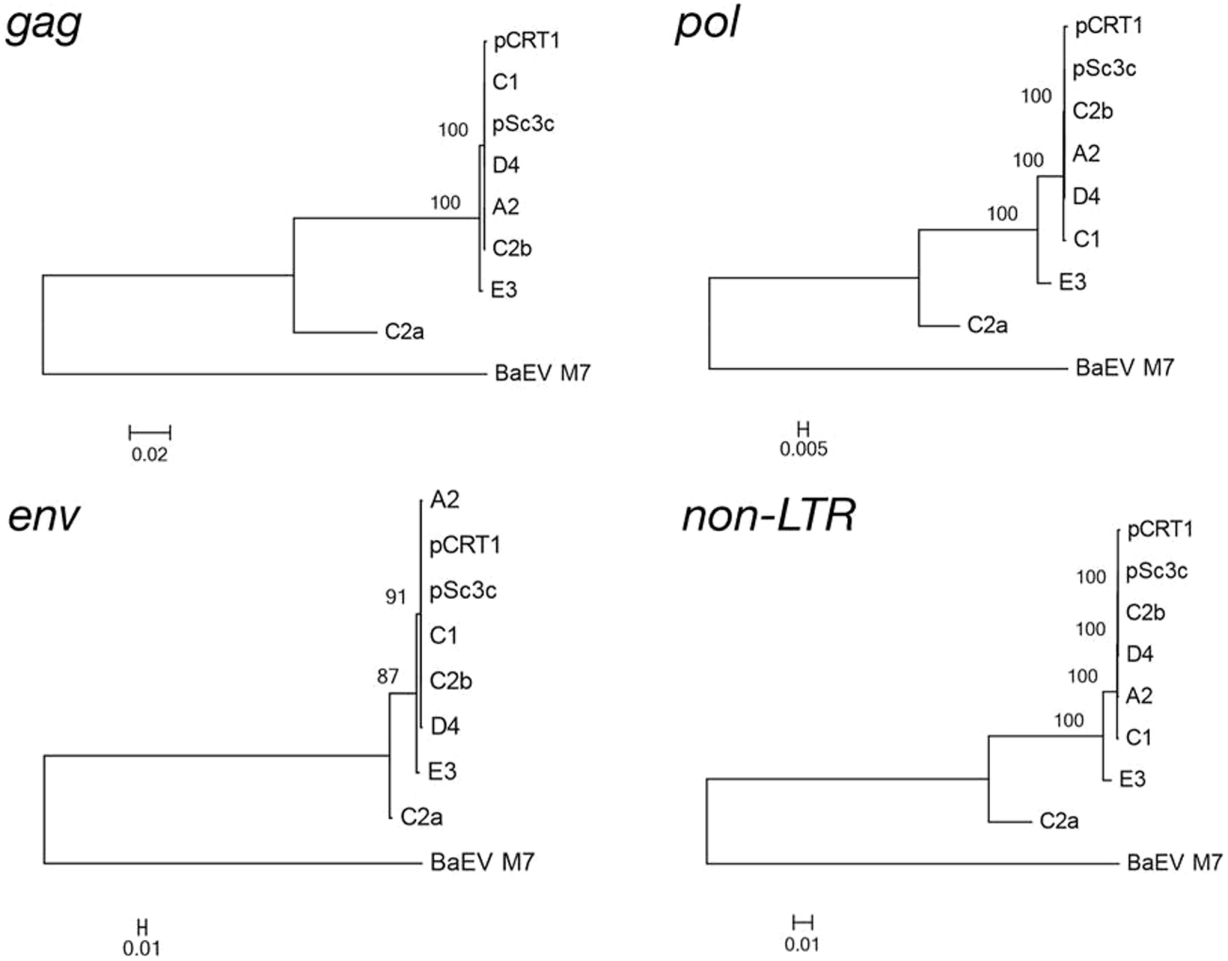
The maximum likelihood phylogenetic trees of RDRSs. We used nucleotide sequences of *gag*, *pol*, *env* and full length excluding LTR. The general time-reversible (GTR) model of nucleotide substitution with the addition of invariant sites (I) and a gamma distribution of rates across sites (Γ) was used to infer the phylogenies. A2, RDRS A2; C2a, RDRS C2a; E3, RDRS E3; D4, RDRS D4; C2b, RDRS C2b; BaEV M7, Baboon endogenous virus strain M7.

**Figure 4:**
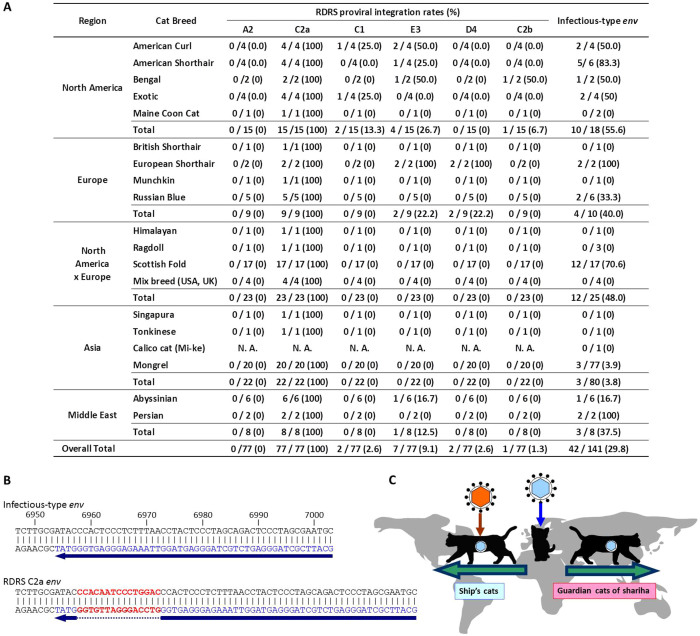
Possession of RD-114 viral infectious-type *env* and RDRSs. (A) Population of RDRS proviruses in feline PBMC. Regions that the cats originally lived in were indicated based on a previous study^40^. (B) Position of reverse primer to detect infectious-type *env* indicated by blue characters and arrowheads. Red characters are the RDRS C2a-specific insertion. Numbers are defined as positions of pSc3c. (C) Cat's journey and process of RDRV integration. Virus particle with blue hexagonal shape and orange hexagonal indicate the oldest RDRV and the new RDRVs, respectively. The picture of cats and the map were drawn by S.S. using the Microsoft PowerPoint.

Firstly, we searched for RD-114 proviral loci in the genome database of domestic cats (Felis_catus_6.2) using the SSEARCH program^18^ with the complete genome sequence of RD-114 strain CRT1 (GenBank accession number: AB559882.1)^19^ as a query; however, we could not identify any proviruses which were identical to the sequences of three previously reported infectious RD-114 virus clones^20^. Instead, we found one defective RDRS locus on feline chromosome C2 and designated it as RDRS C2a (proviruses were named after the chromosome numbers on which they are located). Quite recently, by using the sequences of RD-114 virus infectious clones as queries, Tamazian *et al*. reported that there are twelve RD-114 virus-like elements with more than 50% sequence identity, which cover at least 50% of query sequences in domestic cat genomes^21^. However, their sequences are not available for further analyses. Next, we investigated whether RDRS(s) capable of inducing RD-114 virus are present in all cat genomes by Southern blot analysis. By using *Hin*dIII and a *gag* probe, we detected a 2.6-kb fragment corresponding to the *Hin*dIII fragment of RD-114 virus molecular clones in RD-114 virus producer Crandell-Rees feline kidney (CRFK) cells and RD-114 virus non-producer cell lines such as G355-5 (a feline fetal astrocyte cell line), *Felis catus* whole fetus-4 (fcwf-4) (a feline macrophage-like cell line) and FeT-J (an interleukin-2-independent feline T-cell line) cells (Fig. 1B). By using *Hin*dIII and an *env* probe, we detected a 3.2-kb fragment corresponding to the *Hin*dIII fragment of RD-114 virus molecular clones only in CRFK cells but not RD-114 virus non-producer cell lines (Fig. 1D). In accordance with the previous studies^2^,^14^,^15^, there are polymorphisms present in RD-114 virus-related sequences with a conserved *env* region. By southern blotting hybridization using *Sac*II, *Eco*RI or *Sph*I and probes for *gag*, *pol* and *env* genes, we revealed that RD-114 virus-related *env* copy numbers were less than *gag-pol* copy numbers (Fig. 1B, C, D). Inverse nested PCR targeting RD-114 virus *env* identified three proviruses (designated as RDRS A2, C2a, and C1, respectively) in CRFK cells (Fig. 2 and Fig. S1). We also identified three proviruses (RDRS C2a, E3 and D4) in primary feline peripheral blood mononuclear cells (PBMCs) and two proviruses (RDRS C2a and C2b) in FER cells (feline embryonic fibroblasts that produce RD-114-related virus) (Fig. 2 and Fig. S1). Again, these proviruses were not identical to the sequences of infectious RD-114 virus clones. Four of these proviruses (RDRS A2, C1, D4 and C2b) have the entire open reading frames (ORFs) for *env* and two of them (RDRS C1 and C2b) have intact ORFs for all viral genes (*gag*, *pol* and *env*) (Fig. 2). We found that the 3.2-kb *Hin*dIII fragment containing *env* could be generated by digestion of four RDRSs (RDRS A2, C1, D4 and C2b) which have the intact ORF for *env* gene. RDRS C2a of CRFK, G355-5 cells and feline PBMCs (*n* = 2) have stop codons within all viral genes in the same fashion. Phylogenetic analysis of RDRSs and RD-114 virus clones indicated that RDRS C2a belongs to a separated group from the other RDRSs and infectious RD-114 virus clones (Fig. 3). In feline cell lines, although all lines have RDRS C2a, RD-114 viral producer cell lines do not have any other loci in common (Table 1). These data suggest that a single RDRS is not responsible for the production of RD-114 viral particles. We further investigated cat populations for the possession of RDRSs in their genomes. Similar to the data obtained from cell lines, we also found that all cats have RDRS C2a in common, while the possession patterns of the other RDRS loci varied depending on the regions where cats live or breed (Fig. 4A).

### Timelines for acquisitions of RDRSs in feline species

**Figure 5:**
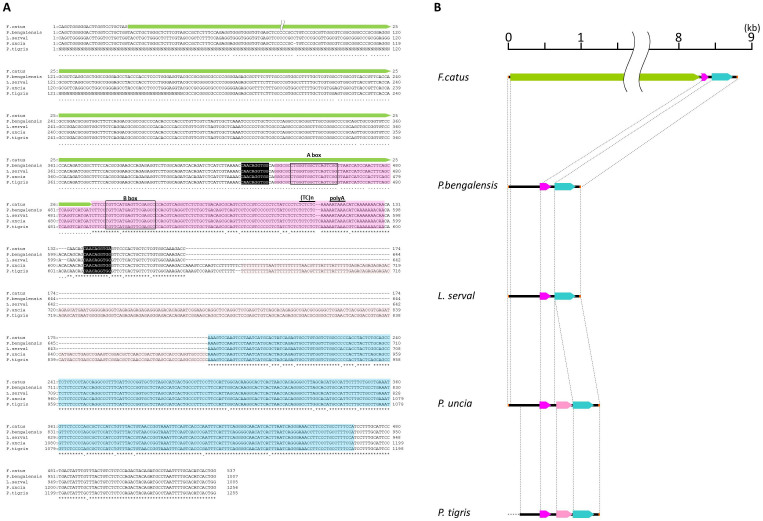
Comparison of sequences of RDRS C2a integration sites in domestic cat's (*Felis catus*) and other Felidae species' genomes. (A) Alignment of sequences of 5′ and 3′flanking regions of RDRS C2a in domestic cat (*Felis catus*) and their corresponding sites' sequences in leopard cat (*P. bengalensis*), Serval cat (*L. serval*), Snow leopard (*P. uncia*) and Tiger (*P. tigris*). Asterisks indicate conserved nucleotides between four species. Target site duplications (TSDs) for SINEC_Fc2-like sequence are shaded in black. (B) Schematic view of RDRS C2a integration site and adjacent sequences of Felidae genomes. Lime green arrow indicates RDRS C2a. Pink and pink-red arrows indicate SINEC_Fc2 and C SINEC_Fc2-like sequence, respectively, and aqua arrow indicates LINE-like sequences. Short orange lines at both sides indicate primer positions used for PCR.

We next estimated the integration time of RDRSs. The divergence accumulated between 5′ and 3′ LTRs over time was previously used as a molecular clock to investigate the integration time of ERV^22^. Thus, we tried to estimate the integration time of the RDRSs by assessing the sequence differences between 5′ and 3′ LTRs. The proposed rate of 1.2 × 10^−8^ substitutions/site/year in cats was adopted for calculating proviral insertion time^23^. Four of the six proviruses (RDRS A2, E3, D4 and C2b) have identical 5′ and 3′ LTRs, which are suggestive of relatively recent integrations into the host genome, less than 0.2 million years ago (MYA), possibly by RDRV infection (Fig. 2C). On the other hand, RDRS C1 and C2a have one and eight nucleotide differences, resulting in an estimated time of integration of RDRS C1 and C2a around 0.2 and 1.6 MYA, respectively (Fig. 2C). These periods are after domestic cats separated from the Sand cat lineage^24^. The time of ERV integration can be also estimated by determining whether phylogenetically closely related animal species share these proviruses in the same genome location. The oldest RDRV provirus C2a was not present in the genome of the Leopard cat (*Prionailurus bengalensis*) (*n* = 4) which inhabits Vietnam. Instead, we found a 54-bp sequence within the same locus. Intriguingly, the 54-bp sequence is part of a SINE element (Repbase ID: SINEC_Fc2), predicted by the Repeatmasker program (http://www.repeatmasker.org/) (Fig. 5). The SINE-like sequence is defined by a tRNA-related region, harboring RNA polymerase III-specific internal promoter with its conserved regulatory elements, A-box and B-box, followed by a microsatellite region (TC)n and by an A/T-rich tail with the polyadenylation signal AATAAA^25^,^26^. The 54-bp sequence in the leopard cat genome contains the A-box, but the domestic cat's genome lacks this part of the SINE element. In other Felidae, including the Serval cat (*Leptailurus serval*), Snow leopard (*Panthera uncia*), and Tiger (*Panthera tigris*) (whole genome shotgun sequences, accession number: NW_006711997.1), the full length SINE elements were maintained in their genomes at the same location as the leopard cat, suggesting that SINEC_Fc2 integrated into the genome of the common ancestor of Felidae species and then RDRS C2a integration occurred, leading to the disruption of a part of the SINE sequence (Fig. 5). Anai *et al*. also reported the absence of the RD-114 viral *env* gene in the Tsushima cat (*Prionailurus bengalensis euptilurus*)^27^,^28^. The domestic cat is estimated to have separated from the leopard cat lineage approximately 6.2 MYA^28^. We hypothesize that the oldest RDRV C2a entered the ancestor of domestic cats from 6.2 to 1.6 MYA before the diversification of cat breeds.

### Integration of RDRS coding for infectious-type *env*

Because RDRS sequences vary depending on where cats live or breed, it is possible that unidentified RDRS loci are present in domestic cat genomes of breeds investigated above. We developed a PCR method to differentiate infectious-type *env* on any loci from the defective C2a-type *env* (Fig. 4B). RDRS C2a has a 15-bp insertion in the *env* coding region, whereas the other RDRSs do not. We designed a reverse primer to step over the region of the 15-bp insertion present in RDRS C2a (Fig. 4B). As a result, we revealed that 40 and 56% of domestic cats in Europe and North America have infectious-type *env*, respectively, whereas almost all domestic cats in Asia do not (Fig. 4A). Asian domestic cats containing infectious-type *env* were mongrel cats and their origins were not identified. Interestingly, none of the Asian cats have any RDRSs harboring infectious-type *env* identified in this study. Similarly, most Scottish fold cats have RDRS harboring infectious-type *env*; however, they are unknown RDRSs. These data may indicate that unidentified RDRS(s) with infectious-type *env* are present in Scottish fold cats. Domestic cats (*Felis silvestris catus*) originate from the African wildcat (*Felis silvestris lybica*), a wild cat living in the Middle East, and then spread over the world to Europe or a different route to Asia^24^ (Fig. 4C). Our results suggested that the newer RDRVs (*i.e.* RDRSs possessing infectious-type *env*) might have infected domestic cats after cats started moving from the Middle East. There is a possibility that some additional loci with the infectious-type *env* were acquired in cats through introgression of newer RDRSs into their genomes by crossbreeding between different feline species or cat breeds^21^,^29^,^30^,^31^. Although the original cat breeds of CRFK, 3201 (feline thymic lymphoma) and FER cells possessing the newer RDRSs are unclear, these cell lines were established in the United States or the United Kingdom^32^,^33^,^34^, suggesting that they originated from cats living in these countries.

### Infectivity of RDRSs

**Figure 6:**
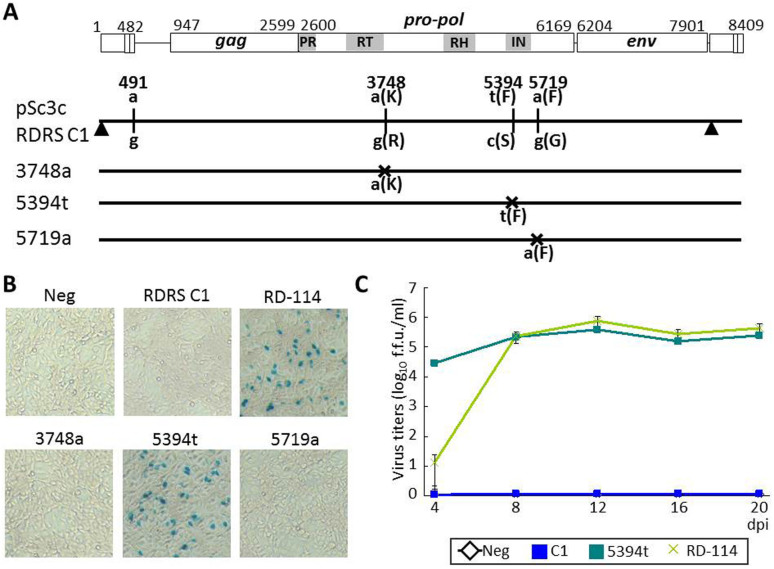
Infectivity of RDRS C1. (A) Differences between RDRS C1 and an infectious molecular clone, pSc3c. Numbers indicate nucleotide locations at pSc3c. Shaded regions are functional domains of *pol* region predicted by Pfam (PR, aspartyl protease; RT, reverse transcriptase; RH, RNaseH; IN, integrase core domain [Pfam accession number: PF00077, PF00078, PF00075 and PF00665, respectively]). Differences between RDRS C1 and pSc3c are indicated by vertical lines (point mutations) and filled triangle (insertion). RDRS C1 mutants have mutations marked by crosses. (B, C) LacZ marker rescue assay performed using TE671 cells as target cells. Infection with LacZ pseudotype viruses (RD-114 virus, RDRS C1 wild-type and RDRS C1 mutants) were visualized by X-Gal staining (B) and the virus titers were expressed as f.f.u./ml (C). Assays were performed in triplicate and the data are shown as the mean viral titers ± standard errors.

Among the RDRS loci, RDRS C1 and C2b have intact ORFs for *gag*, *pol* and *env* genes, and RDRS C1 was most similar to the sequences of infectious RD-114 virus clones^20^. RDRS C1 has five differences, when compared with an infectious RD-114 virus clone, termed pSc3c; LTR has one additional direct repeat (termedΔDR-A), one point mutation at primer binding site (the “a” to “g” change at position 491 of pSc3c), and three point mutations in *pol* gene (“a” to “g” at 3748, “t” to “c” at 5394 and “a” to “g” at 5719) resulting in amino acid substitutions (Fig. 6A). To examine if RDRS C1 encodes infectious RD-114-like virus, we transfected human embryonic kidney (HEK) 293T cells with a plasmid encoding RDRS C1. We then collected culture supernatants at 48 hours post transfection, and inoculated them onto HEK293T cells transduced with the LacZ marker gene [HEK293T(LacZ) cells]. Virus titers produced in the culture supernatants were measured by the LacZ marker rescue assay as described previously^35^. As a result, we found that RDRS C1 was not infectious. We further evaluated the effects of the three point mutations in the *pol* region on the infectivity of RDRS C1. We constructed single mutants, which were made by site-directed mutagenesis^36^. Two mutants were not infectious, whereas one mutant (5394t mutant) with a mutation introduced in the integrase core domain (Pfam accession number: PF00665), predicted by Pfam, (http://pfam.sanger.ac.uk/) had infectivity as an RD-114 virus clone (Fig. 6B and C). From these results, we conclude that the “t” at 5394 is crucial for infectivity for RDRS C1 infectivity. The same mutation in RDRS C1 has also been observed in the sequences of two unrelated cats.

In addition to CRFK cells, FER cells produce infectious RD-114-related viral particles^3^. Because FER cells have RDRS C2b which has intact ORFs for *gag*, *pol* and *env* genes (Fig. 2), we suspected that RDRS C2b may encode an infectious RD-114-like virus. RDRS C2b has a 9-bp insertion at the *pol* coding region, resulting in the insertion of three amino acids in comparison with pSc3c (Fig. S2). We examined the infectivity of RDRS C2b in the same way as described above. Consistent with results of the other RDRSs identified in this study, we found that RDRS C2b was not infectious (Fig. S2).

### Recombination of RDRSs

**Figure 7:**
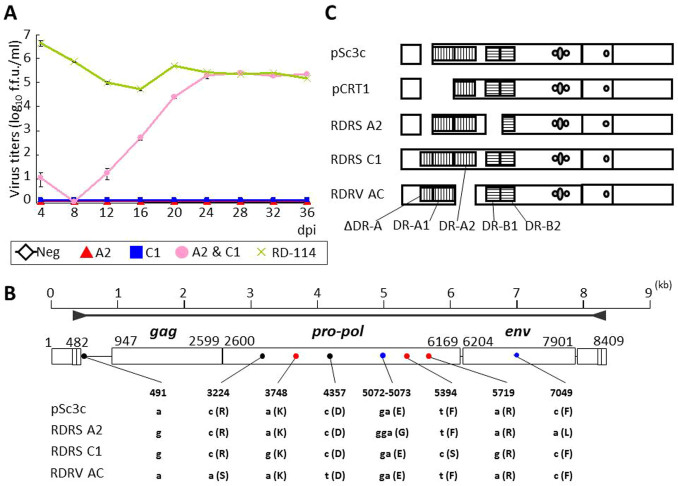
Recombination between RDRS A2 and C1. (A) Growth of RDRV AC in HEK293T cells. The RDRS plasmids were transfected into HEK293T cells and supernatants were inoculated into HEK293T(LacZ) cells, and then virus production was monitored by the LacZ marker rescue assay. Assays were performed in triplicate and the data are shown as the mean viral titers ± standard errors. (B) Comparison of nucleotide sequences of pSc3c, RDRS A2, C1 and RDRV AC virus. The nucleotide positions of pSc3c are shown. Dark grey arrowheads indicate positions of primers used for PCR. Small letters indicate nucleotides. Capital letters in parentheses indicate amino acids. Dots indicate differences between RDRV AC and the other clones. Colors of dots are dependent on where are mutations from (Red, derived from A2; Blue, from C1; Gray, from A2 or C1; Black, undetermined). (C) LTR structures of RD-114 virus clones (pSc3c and pCRT1), RDRS A2, C1 and RDRV AC.

Because we could not find any loci which encode infectious RD-114 virus, we hypothesized that recombination between RDRSs resulted in the production of infectious RD-114 viral particles. We attempted to reproduce the recombination event by transfection of two RDRS plasmids. Because CRFK cells, from which infectious RD-114 virus is produced, have RDRS A2, C2a and C1 loci, we suspected that an infectious RDRV might be generated by recombination between RDRS A2 and C1. We transfected two plasmids encoding RDRS A2 and C1, and succeeded in recovering replication-competent viral particles (Fig. 7A). By comparing sequences of RDRS A2 and C1, and the recombinant RDRV obtained by transfection of RDRS A2 and C1 (hereinafter referred to as RDRV AC), it was suggested that three changes had occurred (two in the middle of *pol* and one in *pol-env* region) (Fig. 7B). Of the two single nucleotide differences between RDRV AC and pSc3c at positions 3224 and 4357, only the “c” to “a” at 3224 results in an amino acid substitution. The amino acid difference was not present in the predicted functional domains of Pol. The length of LTR of RDRV AC was 27 bp shorter than that of the recombination origin RDRS C1 at the repeat region (Fig. 7C). The 27-bp sequence was repeated in the U3 region of LTR in pSc3c, RDRS A2 and C1, and termed direct repeat A1 (DR-A1). Altogether, infectious RD-114 virus-like particles can be generated by recombination between defective RDRS A2 and C1.

## Discussion

The RD-114 virus was originally isolated from human RD cells after passages in fetal cat brains^4^. Because the parent cell line (RD cells) does not contain the RD-114 virus genome, there has been speculation about the origin of the RD-114 virus. In previous studies, restriction endonuclease map analysis and Southern blot hybridization of cat cellular DNA revealed that there are approximately twenty RDRSs in domestic cat genomes but most have a deleted-*env* region^2^,^14^,^15^. The 4.8 kb *Pst*I-digested fragment corresponding to a complete *env* region was detected in all cat DNAs examined as a single copy in the previous study; however, they did not isolate a clone containing a complete RD-114 provirus^14^. We confirmed that the *Pst*I-digested *env* fragments derived from RDRS C2a is similar to infectious RD-114 virus clones, pSc3c and pCRT1, in size (4.7 kb) with high sequence homology (95.7%, data not shown). These data suggest that the single locus in all cat genomes which Spodick *et al*. identified was in fact RDRS C2a and not the complete infectious RD-114 provirus. Another group identified eight RDRSs (Ren 18c, 20a, 8a, 7c, 10a and 6c) in domestic cat genomes^2^ and found that the 1.0-kb *Bam*HI *gag* fragment specific to the RD-114 virus clones was present in cat genomes as a single copy on chromosome B3; however, domestic cats are polymorphic with respect to the presence or absence of this RD-114 virus-related segment^2^,^15^. Although it has been proposed that all domestic cats harbor infectious RD-114 provirus in their genomes, the proviral locus has not been identified even after extensive mining of feline genome databases.

In this study, we identified six RDRSs in domestic cats genomes. These proviruses are not identical to the sequences of infectious molecular clones of RD-114 virus reported previously^20^. RDRS C1, whose sequence is most similar to those of RD-114 infectious clones, could not produce infectious viral particles. The defect was ascribed to a single nucleotide mutation resulting in an amino acid substitution in the integrase core domain which plays a key role in catalysis^37^. The sequences of RDRS LTRs are rich in diversity, suggesting that each RDRS entered the host genome at different time points. Five of the RDRSs (excluding RDRS C2a) have identical 6-bp target site duplications (TSDs) (Table 2) and there were no differences between 5′ and 3′ LTRs in RDRSs A2, C2b and E3. These data suggest that LTR-LTR recombination did not occur and RDRS invasions into cat genomes occurred relatively recently. Except for RDRS C2a, RDRSs have diverse TSDs, indicating that multiplication of RDRSs occurred through viral re-infection but not genome rearrangements. The mechanism of multiple invasions of RDRVs is unknown at present. Generally, it is considered that integration of ERVs into host genomes confers resistance to the infection of exogenous counterparts^38^. However, it is possible that the expression of newly integrated RDRS is tightly regulated in cats, especially in germ-line cells, to allow multiple integrations of RDRV in cats.

Remarkably, number of RDRS proviral copies vary considerably with type of cell lines and cat breeds. All cats have RDRS C2a in common, but all proviral genes (*gag*, *pol* and *env*) are disrupted in RDRS C2a. The other RDRSs have not yet been fixed in domestic cat genomes. Furthermore, we found that most Asian cats are free from new RDRSs harboring infectious-type *env*.

Because domestic cats originated from the African wildcat living in the Middle East (*Felis silvestris lybica*)^24^, we propose the following scenario (Fig. 4C) for RDRV integration into domestic cat genomes in Europe and Asia. (i) Firstly the ancestral virus of RDRS C2a (RDRV C2a) infected the domestic cat progenitor. (ii) The African wildcats were domesticated in the Middle East where agriculture started around 10,000 years ago. (iii) Then, some domesticated cats moved to Europe and America as ship's cats with Vikings or traders, and became common throughout Europe^39^. Ship's cats could travel to different harbors for chance encounters with wildcats for interbreeding^39^, diversifying the genomes of their offspring but at the same time putting themselves at risk for new RDRV infection and subsequent integration by infectious-type RDRSs. (iv) The other cats went to Asia as guardians of Buddhist shariha along well-established trade routes, termed the Silk Road^39^. With no native wildcats that lived near this road to interbreed with, Oriental domestic cats soon began evolving in their own way^39^ without the risk of infection by new RDRVs.

British Shorthair (BSH) was established in England from European Shorthair which first appeared in Sweden, and later American Shorthair (ASH) and American Curl (a variant of ASH) originated from BSH^40^. Some of these cat breeds have RDRS E3 but others do not. These data indicate that RDRS E3 would be a useful genetic marker to trace the footsteps of these breeds from Europe to America. In future studies, we will investigate the presence of RDRSs in domestic cats living in various regions as well as closely related wild cats to further map cat migration.

Several feline cell lines including CRFK and FER cells produce RD-114 or RD-114-related viruses, but there are no proviruses identical to previously reported RD-114 viral sequences. Several novel retroviruses have been reported to be generated by the recombination of ERVs^41^,^42^,^43^. Therefore, we hypothesized that RD-114 virus-like particles were generated by the recombination between two defective RDRS proviruses. By co-transfection of two defective RDRS A2 and C1, we demonstrated that infectious RD-114-like viral particles were generated through recombination. The resultant recombinant, RDRV AC, and infectious clones of RD-114 virus were nearly identical in coding sequences; however, the LTR of RDRV AC was not identical to those of the infectious clones, pCRT1 and pSc3c. The changes in LTR length frequently occurred in repeat sequences during cell passages *in vitro* as observed in other gammaretroviruses such as porcine endogenous retrovirus^44^,^45^. FER cells also produce RD-114-related viral particles^3^, despite the lack of RDRS A2 and C1 in their genomes (Table 1). Similar to the case of CRFK cells, we speculated that an RD-114-related virus could have been generated by recombination between RDRS C2b which has the infectious-type *env* and some other unidentified RDRSs or FcEV.

Retroviral recombination events occur by template switching during reverse transcription^46^,^47^. Xenotropic murine leukemia virus-related virus (XMRV) is one such virus which has arisen through the recombination of two defective murine ERVs during passages of human prostate cancer xenografts in immunocompromised nude mice^48^. The defective viruses recombined in human cells but not in mouse cells because the XMRV is xenotropic. Similarly, the first RD-114 virus may have arisen by recombination in human RD xenografts in cat brains^4^,^8^. RD-114-related viruses have also been induced by chemicals in feline cells without xenotransplantation^11^,^12^,^13^, where a RD-114-related virus may have arisen in feline cells without contact with cells from other species. In these cases, defective viral particles co-packaged with RDRS genome infected feline cells and recombined during reverse transcription. Resultant replication-competent viruses may have expanded in the feline cells, because feline cells are permissive to RD-114 virus^3^,^49^. Additionally, we should consider a possibility that RDRS C1 produces infectious viral particles by spontaneous mutation like Emv2 in C57BL mice^50^,^51^,^52^. Emv2 has a defect in the *pol* gene, compromising the ability of the Emv2 to produce infectious virus particles^53^. However, Emv2 can produce an infectious endogenous ecotropic murine leukemia virus (E-MLV) through the acquisition of a spontaneous mutation in the *pol* gene *in vivo* in aging C57BL mice^50^ or by backcrossing between C57BL and E-MLV-negative mice^51^. Therefore, we cannot exclude the possibility that RDRS C1 produces infectious viral particles through spontaneous mutations *in vivo*.

In the previous study, we revealed ubiquitous expression of the RD-114 viral receptor, termed ASCT2, in domestic cat tissues^49^, suggesting that RD-114 viral recombination events may occur in cats, and the infectious RD-114 virus may be generated in some groups of cats having RDRSs harboring infectious-type *env*. Recently, it was reported that in immune-compromised mice, ERVs in the host genome were resurrected and induced lymphoma^54^,^55^. In these cases, receptors for infectious ERVs are expressed in tissues of mice and infectious ERVs re-infected the host cells and induced lymphoma possibly via insertion of the ERVs in the vicinity of cellular proto-oncogenes^54^,^55^. In this study, most RDRSs are not yet fixed in cat genomes, but some of them have the potential to generate a recombinant virus which could re-infect the host cells. This recombinant virus may re-infect cat cells which do not express RDRS Env, leading to new integrations. Some of the new integrations may induce diseases in cats, if the integration occurred in the vicinity of proto-oncogenes. While it is still unknown whether RD-114 virus causes any diseases in cats, our report that RD-114 virus can be generated by recombination will be useful for further investigation of the pathogenicity of this virus.

## Methods

### Ethics statement

All animal care and procedures that were performed in this study conformed to the guidelines for animal experiments at Institute for Virus Research in Kyoto University (IVRKU), and all experimental protocols were approved by the Committee on the Ethics of Animal Experiments of IVRKU. Blood samples of domestic cats were collected for diagnostic purpose in veterinary clinics. Genomic DNAs of Leopard cats, serval and snow leopard were stored samples which had been used in our previous studies^56^,^57^.

### Blood and tissue samples

Domestic cat blood samples for isolation of PBMCs were provided from Fujimura Animal Hospital (Osaka, Japan), Kyoritsu Seiyaku Corporation (Tokyo, Japan) and Veterinary Clinics in Japan. The Snow leopard and Serval cat tissue samples were provided from Sapporo Maruyama Zoo (Hokkaido, Japan). These animals died from feline parvovirus infection^57^.

### Cell cultures

FeT-J and 3201 cells were cultured in RPMI 1640 medium (Sigma-Aldrich, Tokyo, Japan) supplemented with 10% heat-inactivated fetal calf serum (FCS), penicillin (100 IU/ml) and streptomycin (100 μg/ml) (Invitrogen, Carlsbad, CA). CRFK, G355-5, fcwf-4, FER, AH927 (feline embryonic fibroblast), HEK293T and TE671 (human rhabdomyosarcoma) cells were cultured in Dulbecco's modified Eagle's medium (DMEM) (Sigma-Aldrich) supplemented with 10% FCS, penicillin (100 IU/ml), and streptomycin (100 μg/ml) (Invitrogen).

### Genomic DNA isolation and genomic PCR

Genomic DNA was extracted from cell lines and PBMCs using a QIAamp DNA Blood Mini Kit (QIAGEN, Valencia, CA). Genomic PCR was performed by using PrimeSTAR GXL polymerase (TaKaRa, Shiga, Japan) or Ex Taq polymerase (TaKaRa). The former was used for cloning RDRSs and the recombinant RDRV, and the later was used for screening for the presence of RDRSs. Amplicons were analyzed by electrophoresis in 0.8% or 2% agarose gels. Primers used in this study are listed in Table S1.

### Southern blotting

A total of 5 μg (or 3 μg) genomic DNA was isolated from PBMCs or cell lines, digested with *Hin*dIII for detection of *gag* and *env* genes, *Sac*II for detection of *gag* and *pol* genes, *Eco*RI for detection of *env* genes, or *Sph*I for detection of *gag* and *env* genes. Digested fragments were separated by a 1.0% agarose gel and transferred to positive charged-nylon membrane (Roche Diagnostics, Indianapolis, IN). Hybridization was performed using digoxigenin (DIG)-labeled PCR probes and DIG easy Hyb (Roche) at appropriate temperatures (50°C for *gag* and *pol*, 65°C for *env* detection) for 16 hours. After hybridization, the membrane was washed with a low stringency buffer (0.1% SDS and 2 × SSC) at room temperature followed by a high stringency buffer (0.1% SDS and 0.1 × SSC) at 68°C. The hybridized bands were visualized using CDP-Star reagent (Roche) following the manufacturer's instructions. Probes were prepared from an infectious molecular clone of RD-114 virus, termed pCRT1^19^, using DIG-labeled PCR probe synthesis kit (Roche) according to the manufacturer's instructions with primers used listed in Table S1.

### Inverse PCR

Genomic DNA was digested with *Eco*RI or *Sph*I and 100 ng of digested genomes were ligated by T4 ligase (TOYOBO, Osaka, Japan) at 16°C overnight. Ligated DNA was amplified with specific primers listed in Table S1 using PrimeSTAR GXL polymerase (TaKaRa). Second PCR products were cloned into a pCR4 Blunt TOPO vector (Invitrogen) and sequenced. Loci of RDRSs were determined by comparing flanking sequences with the genome database of domestic cats (Felis_catus_6.2).

### Cloning of RDRSs

Full-length RDRSs were amplified using specific primers (listed in Table S1) on flanking regions and amplicons were cloned into pCR BluntII TOPO vector (Invitrogen).

### Infection assay

HEK293T cells were transfected with 4 μg of RD-114 virus molecular clones or plasmids containing RDRSs using Lipofectamine2000 (Invitrogen). The pcDNA3.1 vector was used for mock transfection. Infectivities of RDRSs were examined by the LacZ marker rescue assay^28^. Culture supernatants were collected 48 hours post-transfection and filtrated through 0.45 μm-pore-size filters and then inoculated onto HEK293T(LacZ) cells with 8 μg/ml polybrene. After inoculation, supernatants were collected every four days. TE671 cells were seeded in 96-well plates at 1 × 10^4^ cells per well one day before infection and diluted supernatants of inoculated HEK293T(LacZ) cells were inoculated onto TE671 cells in the presence of 8 μg/ml polybrene. Two days after inoculation, the inoculated cells were stained with X-Gal, and virus titers, expressed as focus forming units (f.f.u.)/ml, were determined as described previously^35^,^47^. Assays were conducted in triplicate for each individual sample.

### Preparation of RDRS C1 mutants

RDRS C1 mutants were made by *Dpn*I method^36^. PCR was performed using PrimeSTAR GXL Polymerase (TaKaRa) with primers listed in Table S1. RDRS C1 plasmid DNA was used as template. Amplicons were digested with *Dpn*I to eliminate the parental plasmid DNA. Digested DNA fragments were purified and transformed into *Escherichia coli* DH5 alpha.

### RDRV AC isolation

HEK293T cells were transfected with 4 μg of pCRT1 or plasmids containing RDRSs using Lipofectamine2000 (Invitrogen). pcDNA3.1 vector (Invitrogen) was used for mock transfection. Culture supernatants were collected 48 hours post-transfection and filtrated through 0.45 μm-pore-size filters and then inoculated into HEK293T cells with 8 μg/ml polybrene (Sigma-Aldrich). After inoculation, supernatants were collected and filtrated every four days. TE671 cells were seeded in 6-well plates at 1 × 10^6^ cells per well one day before infection and diluted supernatants of the inoculated HEK293T cells (36 d.p.i.) were inoculated onto TE671 cells in the presence of 8 μg/ml polybrene. Six hours after inoculation, genomic DNAs were extracted and proviruses were amplified by PCR. PCR was performed with PrimeSTAR GXL polymerase (TaKaRa) and primers listed in Table S1, according to the manufacturer's instructions. The forward primer was designed in the R region of 5′LTR and reverse primer was designed in the U5 region of 3′LTR (positions of primers were indicated in Fig. 7).

### Sequence Analyses

Sequencing was performed by a commercial DNA sequencing service (FASMAC, Kanagawa, Japan). Alignment of nucleotide sequences was computed using L-INS-i program in MAFFT version 7^58^, and their phylogeny was inferred by RAxML version 7.2.6 that is based on the maximum likelihood method^59^.

### GenBank accession number

RDRS A2, C2a, C1, E3, D4 and C2b are deposited in GenBank as LC005744- LC005749.

## Author Contributions

S.S. and T.M. designed the experiments. S.S. performed the experiments. S.S., S.N. and T.M. analyzed data and wrote the manuscript. All authors read and approved the final manuscript.

## Supplementary Material

Supplementary Information Supplementary Information

## Figures and Tables

**Table 1:** Population of RDRS proviruses in feline cell lines

		RDRS proviral integration							
Origin	Cells	A2	C2a	C1	E3	D4	C2b	Infectious-type *env*	Viral production
kidney	CRFK	+	+	+	-	-	-	+	+
glial	G355-5	-	+	-	-	-	-	-	-
whole fetus	fcwf-4	-	+	-	-	-	-	-	-
T lymphocyte	FeT-J	-	+	-	-	-	-	-	-
lymphoma	3201	-	+	+	+	-	-	+	-
Embryo fibroblast	FER	-	+	-	-	-	+	+	+
	AH927	-	+	-	-	-	-	-	-
Total integration rates (%)		1/7 (14.3)	7/7 (100)	2/7 (25.0)	2/7 (28.6)	0/7 (0)	1/7 (14.3)	3/7 (42.9)	

**Table 2:** RDRS proviral target site duplications (TSDs). Flanking 6-bp TSD sequences and loci are shown. U.D. is undetectable

Provirus	Chromosome	TSD sequence	Locus of TSD
RDRS A2	A2	TGATTT	153,290,986-153,290,991
RDRS C2a	C2	U.D.	U.D.
RDRS C1	C1	TGGTAT	59,327,845-59,327,850
RDRS E3	E3	TGATCT	28,850,909-28,850,914
RDRS D4	D4	TGTCTC	77,169,718-77,169,723
RDRS C2b	C2	GGCAGG	111,337,840-111,337,845
