# Supplementary material for: Multiple invasions of an infectious retrovirus in cat genomes
Source: Sci Rep. 2015 Feb 2;5:8164. doi: 10.1038/srep08164 (PMC4313119; doi:10.1038/srep08164)
Supplement: Supplementary Information [file srep08164-s1.pdf]

Supplementary Information

Multiple invasions of an infectious retrovirus in cat genomes

Sayumi Shimode, So Nakagawa & Takayuki Miyazawa

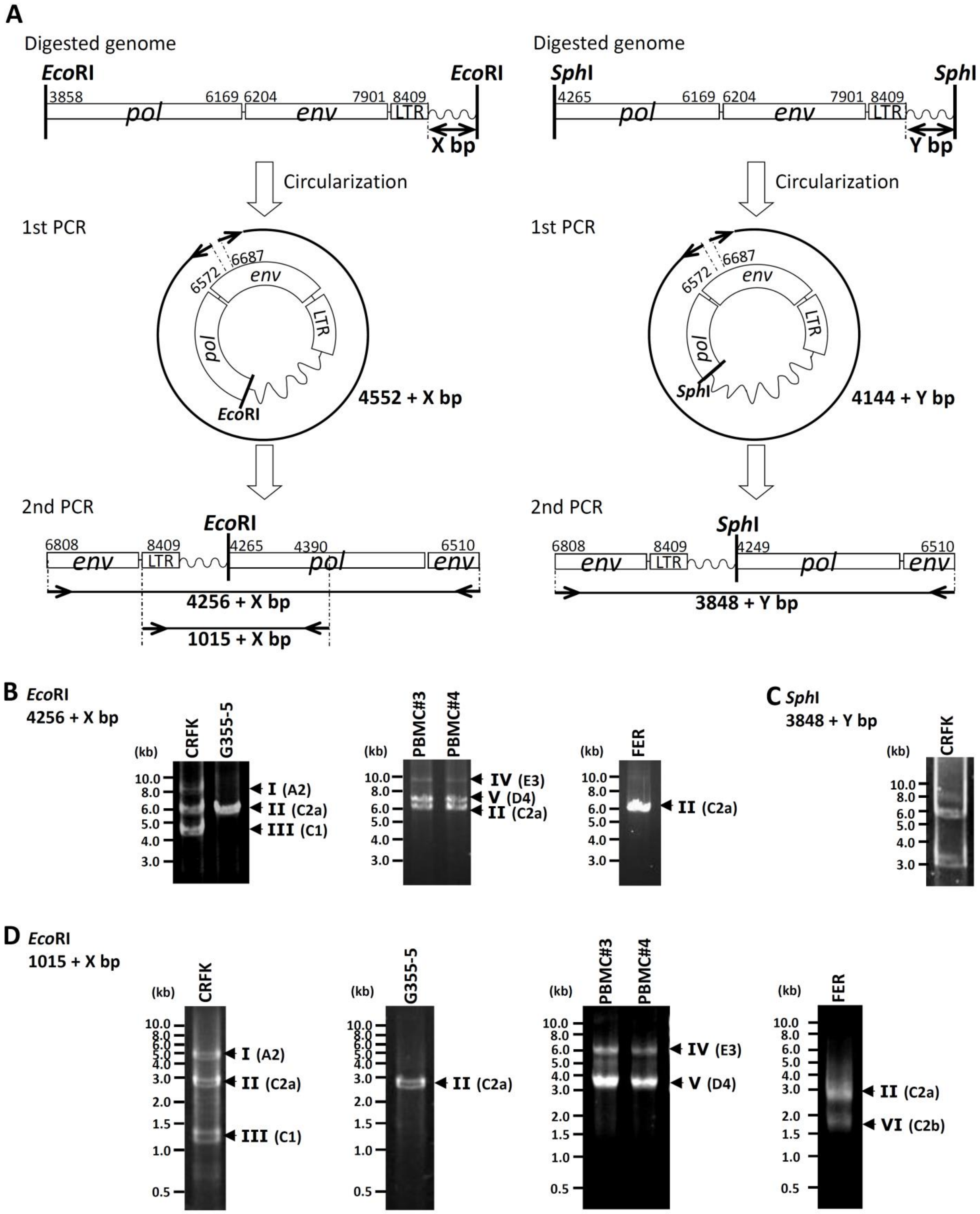

Figure S1 Inverse PCR to identify 3'flanking sequences of RD-114 proviruses in the domestic cat's genome. (A) Genomic DNAs were digested with *Eco*RI (left) or *Sph*I (right), circularized, and then amplified the 3'flanking region using *env* primers (1st PCR). Second PCRs were performed with primers shown in the lower diagrams (2nd PCR). (B-E) Agarose gel electrophoretic profiles of inverse PCR products. Predicted sizes of amplicons were shown under each restriction enzyme. Greek numbers (I-VI) are assigned to discriminate which loci derived from. I, RDRS A2; II, RDRS C2a; III, RDRS C1; IV, RDRS E3; V, RDRS D4; VI, RDRS C2b.

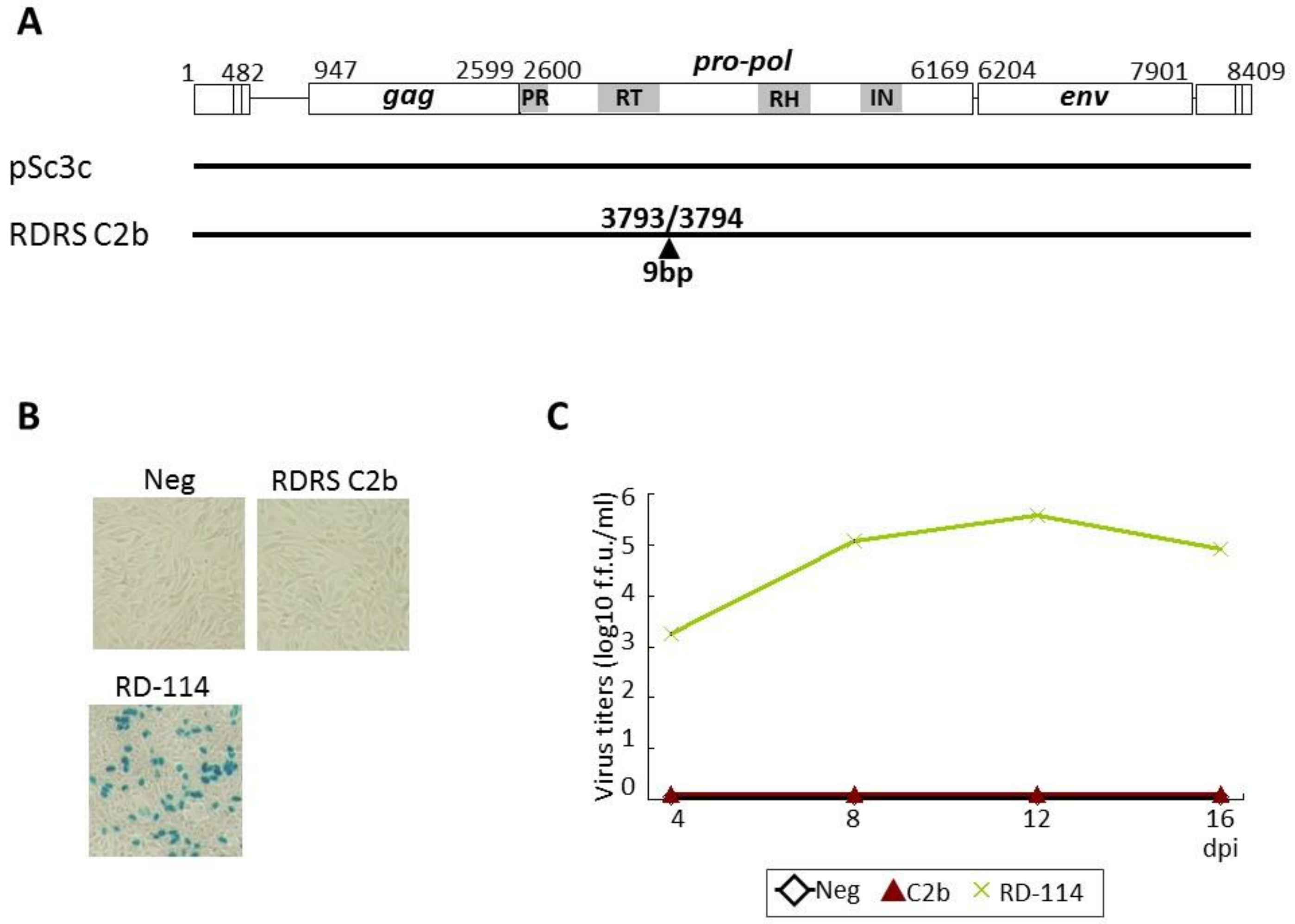

Figure S2 Infectivity of RDRS C2b. (A) Nucleotide differences seen between RDRS C2b and an infectious molecular clone, pSc3c. Numbers indicate nucleotide locations at pSc3c. Shaded regions are functional domains of *pol* region predicted by Pfam (PR, aspartyl protease; RT, reverse transcriptase; RH, RNaseH; IN, integrase core domain [Pfam accession number: PF00077, PF00078, PF00075 and PF00665, respectively]). Differences between RDRS C2b and pSc3c are indicated by vertical lines (point mutations) and filled triangle (insertion). (B, C) LacZ pseudotype virus infection assay performed using TE671 cells as target cells. Infection with LacZ pseudotype viruses (RD-114 virus and RDRS C2b) were visualized by X-Gal staining (B) and the virus titers were expressed as f.f.u./ml (C). Assays were performed in triplicate and the data are shown as the mean viral titers ± standard errors.

assay	target		sequence	position	amplicon size (bp)	reference sequence
cloning	RDRS A2	Forward	5'- CATGGTGATCTCTCCAATCC -3'	A2:154291086-153291105 (-121 to -102)	8582	Felis_catus_6.2
		Reverse	5'- CTGTAACAGACTTTCATAAGAG -3'	A2:153290874-153290895 (+96 to +117)		
	RDRS C2a	Forward	5'- CAGCTGGGGGACTTGGTC -3'	C2:3268287-3268304 (-26 to -8)	8820	Felis_catus_6.2
		Reverse	5'- CCAGTGATGTGCAAAATTAGGC -3'	C2:3259233-3259254 (+489 to +510)		
	RDRS C1	Forward	5'- CCTACTTAGGATGCATGCGC -3'	C1:59327935-59327954 (-111 to -92)	8654	Felis_catus_6.2
		Reverse	5'- GAGCAGTTACAGCATTTACCC -3'	C1:59327769-59327789 (+61 to +81)		
	RDRS E3	Forward	5'- GAGAAAATTCAGAGACGGGG -3'	E3:28850970-28850989 (-82 to -63)	8450	Felis_catus_6.2
		Reverse	5'- GAGAGATCGAGTGAGTGGG -3'	E3:28850800-28850818 (+96 to +114)		
	RDRS D4	Forward	5'- CCCATCATGCTTGTAAGCC -3'	D4:77169653-77169671 (-47 to -72)	8557	Felis_catus_6.2
		Reverse	5'- CTTGTCATAAACTCTAGGCCTG -3'	D4:77169782-77169800 (+57 to +75)		
	RDRS C2b	Forward	5'- CGGGTCTTTGCCATGTAC -3'	C2:111337906-111337923 (-68 to -85)	8593	Felis_catus_6.2
		Reverse	5'- GTAAGAGTGCATGTTCTAGGG -3'	C2:111337755-111337775 (+70 to +90)		
	RDRV AC	Forward	5'- GCTCTCCCGCTTTCTAAC -3'	356-373	8029	AB705392
		Reverse	5'- TGTTAGGAGCCAAACTCCTAGGCC -3'	8386-8409		
screening	RDRS A2	Forward	5'- CCCAACAGGAATGGTCATTTTATG -3'	6212-6235	2282	AB559882
		Reverse	5'- CTGTAACAGACTTTCATAAGAG -3'	A2:153290874-153290895 (+96 to +117)		Felis_catus_6.2
	RDRS C2a	Forward	5'- CCCAACAGGAATGGTCATTTTATG -3'	6212-6235	2261	AB559882
		Reverse	5'- CCAGTGATGTGCAAAATTAGGC -3'	C2:3259233-3259254 (+489 to +510)		Felis_catus_6.2
	RDRS C1	Forward	5'- CCCAACAGGAATGGTCATTTTATG -3'	6212-6235	2301	AB559882
		Reverse	5'- GAGCAGTTACAGCATTTACCC -3'	C1:59327769-59327789 (+61 to +81)		Felis_catus_6.2
	RDRS E3	Forward	5'- CCCAACAGGAATGGTCATTTTATG -3'	6212-6235	2235	AB559882
		Reverse	5'- GAGAGATCGAGTGAGTGGG -3'	E3:28850800-28850818 (+96 to +114)		Felis_catus_6.2
	RDRS D4	Forward	5'- CCCAACAGGAATGGTCATTTTATG -3'	6212-6235	2273	AB559882
		Reverse	5'- CTTGTCATAAACTCTAGGCCTG -3'	D4:77169782-77169800 (+57 to +75)		Felis_catus_6.2
	RDRS C2b	Forward	5'- CCCAACAGGAATGGTCATTTTATG -3'	6165-6188	2288	AB559882
		Reverse	5'- GTAAGAGTGCATGTTCTAGGG -3'	C2:111337755-111337775 (+70 to +90)		Felis_catus_6.2
	infectious *env*	Forward	5'- CCTCAACGAGGTACAGATATTACA -3'	6590-6613	414	AB705392
		Reverse	5'- GCATTCGCTAGGGAGTCTGCTAGGGAGTAGGTTAAAGAGGGAGTGGGTAT -3'	6954-7003		
southern blotting	RD-114 *gag*	Forward	5'- TGGGACAGACATTGACTACTCCTC -3'	948-971	1652	AB705392
		Reverse	5'- CTACTCGCTCTCTTCGCCCAAG -3'	2578-2599		
	RD-114 *pol*	Forward	5'- CAAAAACTCCCCCACTCTCTTTG -3'	3529-3551	1635	AB705392
		Reverse	5'- AGCTTCCGATTACTTAAATGTGT -3'	5141-5163		
	RD-114 *env*	Forward	5'- CCCAACAGGAATGGTCATTTTATG -3'	6212-6235	1689	AB705392
		Reverse	5'- CAATCCTGAGCTTCTTCCTC -3'	7881-7900		
inverse PCR	RD-114 *env*	Forward	5'- CCCATCCATATCTCCGATGGT -3'	6687-6707	4552 + flanking (*Eco* RI)	AB705392
		Reverse	5'- AAGCAAGGTGGCCGTGTAG -3'	6551-6572	4144 + flanking (*Sph* I)	
	RD-114 *env*	Forward	5'- TGCCCAAAGTCAGAGATG -3'	6808-6825	4256 + flanking (*Eco* RI)	AB705392
		Reverse	5'- AAGAACTGTGCATCGAGT -3'	6510-6493	3848 + flanking (*Sph* I)	
	RD-114 *pol* ,3' LTR	Forward	5'- TGAGAAGTCAGAACCCCCCACC -3'	1-22	1015 + flanking	AB705392
		Reverse	5'- GTTCAAGGTAACCGGAGGTC -3'	4371-4390		

Table S1. Primers in this study.

The numbers in parentheses are distances from RDRSs' each end.
